# Estrogen-mediated modulation of sterile inflammatory markers and baroreflex sensitivity in ovariectomized female Wistar rats

**DOI:** 10.20945/2359-4292-2023-0521

**Published:** 2024-11-06

**Authors:** Md. Iqbal Alam, Naba Sami, Aftab Alam, Sheema Wazib, Neha Dhyani, Sher Afghan, Mairaj Ahmed Ansari

**Affiliations:** 1 Jamia Hamdard University Hamdard Institute of Medical Sciences and Research Department of Physiology New Delhi India Department of Physiology, Hamdard Institute of Medical Sciences and Research, Jamia Hamdard University, New Delhi; 2 Coventry University School of Life Sciences Coventry UK School of Life Sciences, Coventry University, Coventry, UK; 3 Jamia Hamdard School of Chemical & Life Sciences Department of Biotechnology New Delhi India Department of Biotechnology, School of Chemical & Life Sciences, Jamia Hamdard, New Delhi

**Keywords:** Cardiovascular diseases, baroreflex sensitivity, oxidative stress, inflammatory cytokines, ovariectomy, estrogen

## Abstract

**Objective:**

This study aims to explore the role of estrogen in providing cardioprotective benefits to premenopausal women, examining how hormonal differences between sexes influence the prevalence of cardiovascular diseases (CVDs) in women.

**Materials and methods:**

Eighteen female Wistar rats were equally distributed into three treatment groups. Animals in Group I (sham-operated) and Group II (ovariectomized [OVX]) received oral saline solution at a dose of 2 mL/kg. Group III (OVX+E_2_) received oral E_2_ 2 µg/mL/kg after ovariectomy. Hemodynamic parameters and baroreflex sensitivity were determined in all groups. Plasma levels of malondialdehyde (MDA), superoxide dismutase (SOD), and nitric oxide (NO) were measured, along with those of the inflammatory markers tumor necrosis factor-alpha (TNF-α), interleukin-6 (IL-6), and high mobility group box-1 (HMGB-1).

**Results:**

The OVX group, compared with the sham-operated group, displayed significantly altered hemodynamic parameters and baroreflex sensitivity, along with elevated MDA levels and decreased SOD and NO levels. This group also had higher levels of inflammatory cytokines than the sham-operated group. In the absence of estrogen, these factors led to the advancement of cardiovascular abnormalities. In the OVX+E_2_ group, estrogen treatment considerably improved baroreflex sensitivity and hemodynamic profile while reducing the expression of inflammatory cytokines compared with the OVX group, demonstrating anti-inflammatory actions of estrogen.

**Conclusion:**

Estrogen mediates cardioprotection by improving baroreflex sensitivity in ovariectomized Wistar rats through modulation of the NO pathway.

## INTRODUCTION

Cardiovascular diseases (CVDs) remain the leading cause of mortality across the globe and greatly increase expenses for the healthcare system (1). An estimated 17.5 million people died from CVDs in 2012, representing 31% of all global deaths. However, there are differences in CVD incidence among men and women, with hormonal variations providing cardioprotective advantages in the female sex contributing to this variance. This sexual dimorphism is associated with a reduced incidence of CVDs (*e.g.*, stroke, hypertension, atherosclerosis, myocardial infarction) in women compared with age-matched men (2). Young ovariectomized women and postmenopausal women have an increased incidence of CVDs compared with women with intact or functional ovaries, highlighting the importance of sex hormones in cardioprotection (3,4). In premenopausal women, ovarian hormones remain at adequate levels during the menstrual cycle, thereby offering cardioprotective effects (2). However, at the end of reproductive life, the ovaries are no longer able to produce a sufficient amount of female hormones, increasing the risk of CVD in postmenopausal women (3-5). Notably, hormone replacement therapy has been shown to prevent CVDs effectively in postmenopausal women (3).

Results from the Study of Women's Health Across the Nation (SWAN), the Study on Hypertension Prevalence in Menopause in the Italian Population (SIMONA), and the Hormone Estrogen/Progestin Replacement Study (HERS) have shown that hormone replacement therapy is efficient in reversing cardiovascular complications in postmenopausal women (5-7). Hormone replacement therapy is administered using either estrogen alone or a combination of estrogen and progesterone. Even treatment with estrogen alone at moderate doses is associated with a decreased risk of CVD in postmenopausal women (8). Research shows that the main circulating female hormone – estrogen – is cardioprotective through a plethora of mechanisms. The main female hormone and the most abundant form of circulating estrogen is 17-beta-estradiol (E_2_), a complex hormone with pleiotropic effects. In addition to maintaining the functions of sexual organs, E_2_ plays an important role in normal tissue development and several physiological processes, including bone development and skin health (9). It has also a major impact on the vasculature, by modulating inflammation and expression of adhesion proteins (10,11), and a wide range of effects on the heart, including prevention of apoptosis and reduction of damage in cardiac ischemia and reperfusion. Moreover, E_2_ protects the heart (and the intact heart) from ischemia-related injury (11). However, the exact mechanisms by which E_2_ exerts cardioprotection remain unknown.

Nitric oxide (NO), a signaling molecule generated by the NO synthase (NOS) family – neuronal NOS (nNOS), endothelial NOS (eNOS), and inducible NOS (iNOS) – plays a wide range of physiological and pathophysiological roles in the human body and in tumor biology (12,13). For example, NO contributes to protecting against the onset and progression of CVD, hypercholesterolemia, hypertension, and diabetes. The cardioprotective roles of NO include regulation of blood pressure (BP) and vascular tone, inhibition of platelet aggregation, leukocyte adhesion, and prevention of smooth muscle cell proliferation, among others (14). Although it is unclear whether reduced NO bioavailability is the source or the result of endothelial dysfunction, it is thought to be one of the key contributing factors in CVD. Any change in NO bioavailability results in a loss of cardioprotective effects and, in some cases, may even contribute to disease progression (15). Given the pleiotropic effects of NO, it is likely that NOS has an important role in conferring protection in female cardiomyocytes.

Abnormal BP levels are strong predictors of CVDs. A study by Gu and cols. (16) observed a strong, linear, and independent association between BP levels and CVD risk. Baroreflex is an important neural feedback mechanism of BP regulation (17), and impairment of the baroreflex mechanism often accompanies CVDs. The baroreflex sensitivity, an index quantifying the baroreflex's influence on heart rate (HR), has been recognized as an early marker of autonomic control over circulation. Additionally, reduced baroreflex sensitivity can be the cause and/or consequence of autonomic imbalance associated with several CVDs (18).

Based on these considerations, the present study was conducted to explore the mechanisms by which estrogen mediates cardioprotection through the modulation of oxidative stress, inflammation, and autonomic balance, and to examine the relationship between estrogen and NO in ovariectomized female rats. Specifically, our objective was to observe the effects of an oral dose of 17-beta estradiol valerate on baroreflex sensitivity, hemodynamics, oxidative stress markers, and inflammatory markers in these experimental animals.

## MATERIALS AND METHODS

Female Wistar rats (250-300 g, 15-20 weeks) were obtained from the central animal house facility of Hamdard University in New Delhi with permission from the Committee for the Purpose of Control and Supervision of Experiments on Animals (CPCSEA). All experiments were performed according to the Institutional Animal Ethics Committee (IAEC) rules. During the experimental study period, the rats were housed at constant room temperature (22 ± 5 ^o^C), humidity (40%-70%), and light-dark (12-hour light, 12-hour dark) cycles. Food (pellets commercially procured) and water were provided *ad libitum*, unless otherwise indicated.

### Hormone and chemicals

The following hormone and chemicals were used in the experiments: 17-beta estradiol valerate tablets (Intas Pharmaceuticals Ltd., Gujarat, India); phenylephrine (P1240000), sodium nitroprusside (1614501), and BSA (A2153;) – all from Sigma-Aldrich (MO, USA); mouse monoclonal anti-HMGB1 antibody (ab77302), mouse monoclonal anti-TNF antibody (ab1793), and rabbit polyclonal anti-IL6 antibody (ab6672) – all from Abcam (Waltham, MA, USA); HRP conjugated secondary antibodies (anti-mouse [62-6520] and anti-rabbit [65-6120]; Invitrogen, Waltham, MA, USA).

### Treatment groups and surgical procedure

The rats were divided into three groups, with 6 rats in each group. The animals in Group I (sham-operated) and Group II (ovariectomized [OVX]) received oral saline solution 2 mL/kg each, while those in Group III (OVX+E_2_) received oral E_2_ 2 µg/mL/kg after ovariectomy.

Before surgery, all animals were weighed and anesthetized with an intraperitoneal injection of ketamine 75 mg/kg and xylazine 10 mg/kg (19).

In the sham-operated group, an incision was made on both sides of the peritoneal cavity, and the wound was closed and left to heal for 2 weeks. During this time, the animals received oral saline solution (2 mL).

In the OVX group, an incision was made on both sides of the peritoneal cavity, and the ovaries were located and exteriorized with gentle retraction. Both ovaries were removed along with a varying amount of fat after the ovarian arteries were ligated. The wound was sutured and allowed to heal for 2 weeks.

In the OVX+E_2_ group, the animals underwent the same procedure as in the OVX group and, after 2 weeks, received oral estradiol valerate 2 µg/mL/kg for 1 week.

### Measurement of hemodynamic parameters

The rats in all groups were anesthetized using an intraperitoneal injection of ketamine 75 mg/kg and xylazine 10 mg/kg (19). On the 15th day after surgery, each animal's femoral artery was cannulated with a polyethylene catheter attached to a pressure transducer (MLT0699-DC-06A) and to a Power Lab Data Acquisition System for measurement of BP levels (LabChart, version 8.1.8, ADInstruments, Colorado Springs, CO, USA). After the experiment, the recorded BP data were used to calculate the systolic BP (SBP), diastolic BP (DBP), mean arterial pressure (MAP), and HR.

### Baroreflex sensitivity measurement

The baroreflex sensitivity was tested as previously described by Dhyani and cols. (20). A polyethylene catheter was used to cannulate a jugular vein on one side and graded bolus doses of the vasoconstrictor phenylephrine (20-40 mg/mL/kg) and the vasodilator sodium nitroprusside (20-40 mg/mL/kg) were administered. The resulting changes in HR and SBP levels were measured at various time intervals. Data from individual animals were subjected to regression analysis to determine the association between increased SBP or tachycardia elicited by phenylephrine and decreased SBP or associated bradycardia elicited by sodium nitroprusside. A regression coefficient (slope of the regression line) was used to calculate the baroreflex sensitivity in beats per minute per millimeter of mercury (bpm/mmHg).

### Estimation of malondialdehyde

Malondialdehyde (MDA) levels above a certain threshold were measured to assess oxidative stress. As a persistent lipid peroxidation product, MDA was measured following the Ohkawa and cols. (21) method using thiobarbituric acid. Subsequently, phosphate buffer (0.1M, pH 7.4) 0.58 mL, ascorbic acid (100 mM) 0.2 mL, and ferric chloride (100 mM) 0.02 mL were added to 0.05 mL of serum sample. For 1 hour, the reaction mixture was incubated in a water bath at 37 °C. The reaction was stopped by adding 10% trichloroacetic acid 1 mL and 0.67% thiobarbituric acid 1 mL to each tube, and the tubes were then placed in a boiling water bath for 20 minutes before being switched to a crushed ice bath and centrifuged for 10 minutes at 2,500 rpm. The optical density of the supernatant was determined at 532 nm (22) and the MDA equivalents were measured in µmol/mg of protein.

### Estimation of superoxide dismutase

The inhibitory effects of the enzyme superoxide dismutase (SOD) on the auto-oxidation of pyrogallol were used to estimate free radicals (23). Reaction mixtures were prepared using different concentrations of standards, tests, and controls. The mixtures consisted of 100 µL of EDTA and 1 mmol/L of diethylene triamine pentetic acid (DTPA) added to air-equilibrated Tris-HCl buffer (50 mmol/L, pH 8.2), with the final volumes adjusted to 3 mL. Subsequently, 100 µL of standards (with varying SOD amounts) or test sera were added to the reaction mixtures. To obtain unconstrained auto-oxidation of pyrogallol, neither the test sample nor the standard was added to the assay mixture in the controls. To initiate the reaction, 100 µL of pyrogallol (0.2 mmol/L) was added to each vial. The reaction was monitored spectrophotometrically by measuring the rate of absorbance change at 420 nm at every 10-second interval for 4 minutes. The percentage inhibition in the standards and test samples was estimated using the average change in absorbance per minute (24).

### Nitric oxide assay

The rate of oxyhemoglobin conversion to methemoglobin by NO was measured using a Hitachi U-2910 scanning spectrophotometer (Hitachi High-Technologies Corporation, Tokyo, Japan) (25). Plasma samples were combined with Krebs buffer (pH 7.4), 15 mM oxyhemoglobin, 10 mM L-arginine, and 240 nM insulin in a reaction mixture. The mixture was continuously swirled for 45 minutes at 37 °C. The NO content was quantified by measuring the spectral changes in the reaction mixture, specifically the decrease in absorbance at 575 nm and 630 nm, corresponding to the conversion of oxyhemoglobin to methemoglobin. A standard curve was constructed using > 99% pure commercial NO in 0.9% NaCl under identical conditions. The NO quantity was confirmed independently using a chemiluminescence approach.

### Measurement of TNF-α, HMGB-1, and IL-6

Samples were subjected to ELISA to estimate plasma levels of tumor necrosis factor-alpha (TNF-α), high mobility group box-1 (HMGB-1), and IL-6 (26). In a sample test plate, 50 μg of plasma was incubated overnight at 4 °C with an equal volume of coating buffer (0.5 M carbonate buffer, pH 9.6). In the same buffer, 5% BSA was added to inhibit nonspecific binding. The samples were then rinsed in PBS containing 0.05% Tween 20 and incubated in a blocking buffer for 2 hours with diluted (1:500) primary antibodies (TNF-α and IL-6). Subsequently, the samples were rinsed again and treated in the same buffer for 2 hours with diluted (1:2000) secondary antibody-HRP. After washing, the samples were incubated in a carbonate buffer containing 10 mM MgCl_2_ with p-nitrophenyl phosphate 1 mg/mL. Color development was measured at 450 nm. The process was halted by adding 50 µL of 1 M NaOH to the mixture. The results were collected from three different experiments and were expressed as mean ± standard error of the mean (SEM).

### Statistical analysis

Bar graphs and group data of hemodynamics, baroreflex sensitivity, oxidative stress, and expression levels of inflammatory cytokines were plotted using GraphPad Prism, version 9 (GraphPad Software, LLC, San Diego, CA, USA). The data are expressed as mean ± SEM. The statistical significance was assessed using Tukey's test. All tests were two-tailed. Confidence intervals were calculated at a 95% level, and p values < 0.05 were considered significant.

## RESULTS

### Hemodynamics

**Figure 1:**
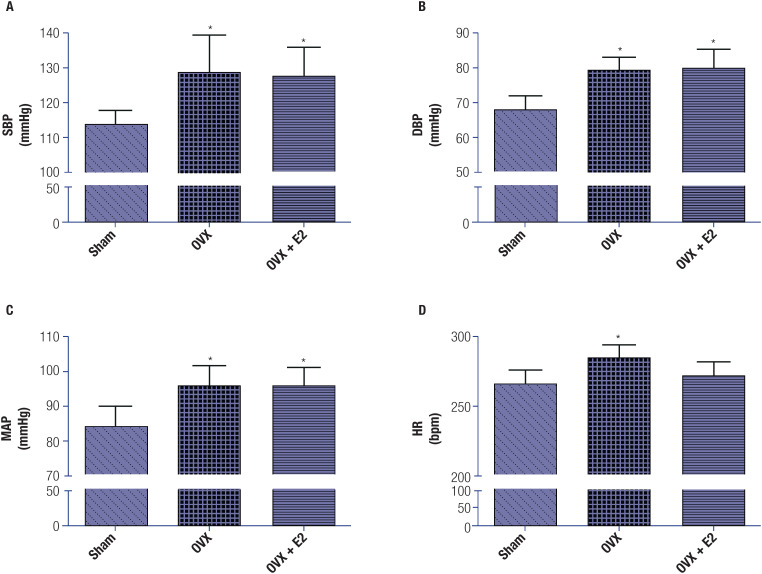
Changes in hemodynamic parameters in the control and test groups (each group included 6 rats). (**A**) Systolic blood pressure (SBP). (**B**) Diastolic blood pressure (DBP). (**C**) Mean arterial pressure (MAP). (**D**) Heart rate (HR). The data are shown as mean ± standard error of the mean. One-way analysis of variance (ANOVA), followed by Tukey's test, revealed significant (*p < 0.05) differences between the sham-operated group and the OVX group. There was a slight improvement with E_2_ treatment in the OVX+E_2_ group compared with the OVX group, although the differences were not significant.

Hemodynamic parameters were markedly altered in the OVX group, as we observed a significant (p < 0.05) increase in BP levels (SBP, DBP, and MAP) and HR in the OVX group compared with the sham-operated group (Figure 1). Treatment with E_2_ slightly improved the altered hemodynamic parameters, as indicated by a decrease in HR and SBP, DBP, and MAP values in OVX+E_2_ rats compared with OVX rats, although the changes were not significant.

### Baroreflex sensitivity

**Figure 2:**
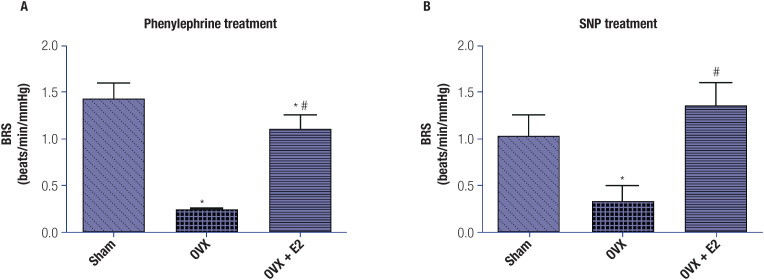
Baroreflex sensitivity (BRS) in all three groups after treatments (each group included 6 rats). (**A**) Response to phenylephrine. (**B**) Response to sodium nitroprusside. The data are shown as mean ± standard error of the mean (SEM). One-way analysis of variance (ANOVA), followed by Tukey's test, revealed significant differences between the sham-operated group and the OVX group (*p < 0.05) and between the OVX group and the OVX+E_2_ group (#p < 0.05). Note: SNP, sodium nitroprusside.

Animals in the OVX group showed a significant (p < 0.05) reduction in baroreflex sensitivity in response to phenylephrine and sodium nitroprusside when compared with the animals in the sham-operated group. Treatment with E_2_ in the OVX+E_2_ group, compared with the OVX group, significantly improved baroreflex sensitivity (Figure 2).

### Oxidative stress

**Figure 3:**
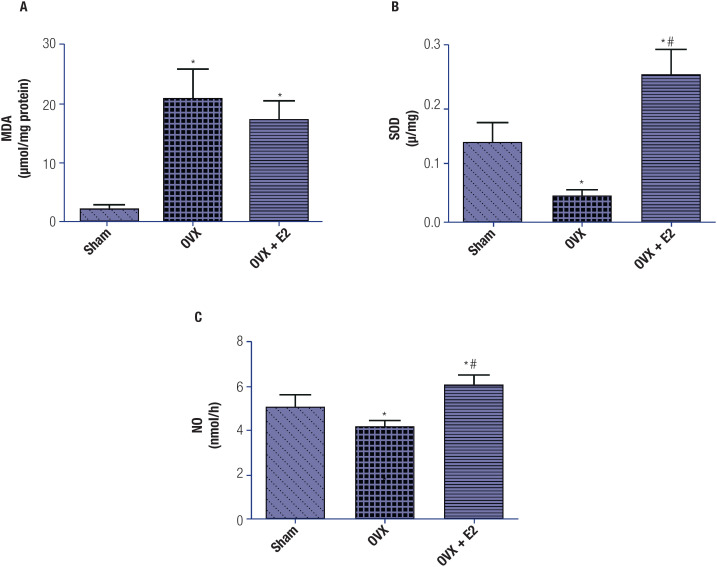
Assessment of oxidative stress in all three groups (each group included 6 rats). (**A**) Malondialdehyde (MDA) levels. (**B**) Superoxide dismutase (SOD) levels. (**C**) Nitric oxide (NO) levels. The data, shown as mean ± standard error of the mean, were obtained from one experiment representative of three independent experiments, all performed in triplicate. One-way analysis of variance (ANOVA), followed by Tukey's test, revealed significant differences between the OVX+E_2_ group and the sham-operated (*p < 0.05) and OVX (#p < 0.05) groups.

In OVX rats, there was a significant increase in plasma MDA levels and significant decreases in plasma SOD and NO levels compared with sham-operated rats. These results reflect a state of oxidative stress in OVX rats. Treatment with E_2_ significantly decreased plasma MDA levels. Additionally, plasma SOD and NO levels increased significantly in the OVX+E_2_ group compared with the OVX and sham-operated groups, suggesting an antioxidative action of E_2_ (Figure 3).

### Inflammatory response

The plasma level of the proinflammatory cytokine TNF-α was significantly elevated (p < 0.001) in OVX rats compared with sham-operated rats. After treatment with E_2_, TNF-α levels reduced significantly (p < 0.001), reaching levels comparable to those in sham-operated animals, thus confirming the anti-inflammatory effects of E_2_.

**Figure 4:**
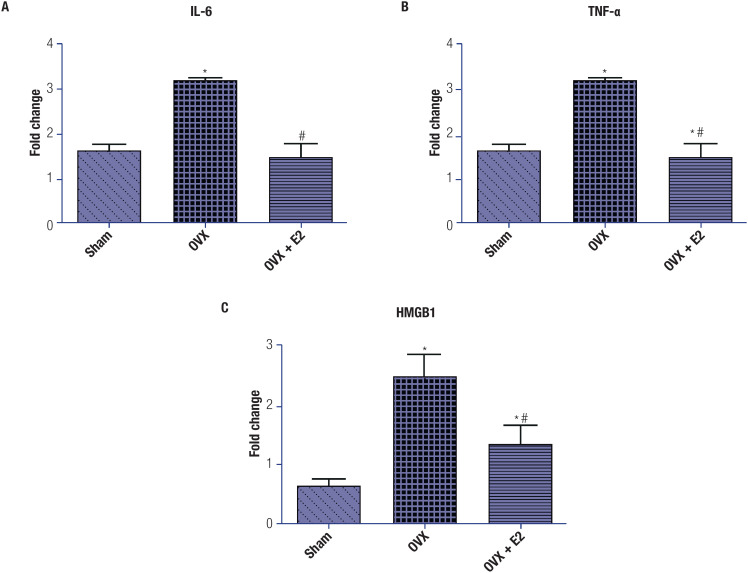
Expression of inflammatory cytokines in plasma samples collected after each treatment. Each group included 6 rats. (**A**) interleukin (IL)-6. (**B**) Tumor necrosis factor-alpha (TNF-α); (**C**) High mobility group box-1 (HMGB-1). The data, shown as mean ± standard error of the mean, were obtained from one experiment representative of three independent experiments, all performed in triplicate. One-way analysis of variance (ANOVA), followed by Tukey's test, revealed significant differences between the OVX+E_2_ group and the sham-operated (*p < 0.05) and OVX (#p < 0.05) groups.

Similar to the TNF-α response, the IL-6 and HMGB-1 levels also increased significantly in OVX rats compared with sham-operated rats. Treatment with E_2_ (in OVX+E_2_ rats) significantly reduced the levels of both IL-6 and HMGB-1. These results further confirm the anti-inflammatory effects of E_2_ (Figure 4).

## DISCUSSION

The results of our study demonstrated the role of estrogen in mediating cardioprotection in ovariectomized Wistar rats. Our model simulated a condition of deficiency of ovarian hormones, which we obtained by submitting the rats to bilateral ovariectomy, allowing us to evaluate hemodynamic (SBP, DBP, MAP, HR) and biochemical (MDA, SOD, TNF-α, IL-6, and HMGB-1) changes. We also observed the animals’ baroreflex sensitivity to evaluate their autonomic balance. To perform these analyses, we assigned the rats to one of three groups, Groups I (sham-operated), Group II (OVX), and Group III (OVX+E_2_).

The results of our experiments indicated that the animals in the OVX group had a significant increase in hemodynamic parameters (*i.e.*, HR, BP, and MAP values) compared with the animals in the sham-operated group. Notably, a previous study has also found that OVX rats have significant increases in BP values and sympathetic activity and that these effects can be attenuated by estrogen replacement (27). Although we found that these parameters slightly improved after E_2_ treatment (OVX+E_2_ group), the improvement was not significant (Figure 1).

The baroreflex sensitivity, an indicator of autonomic control of cardiovascular functions, is widely recognized as a clinical diagnostic tool for assessing individuals with CVD (28). In our study, the baroreflex sensitivity was decreased in OVX rats compared with sham-operated rats (Figure 2). Elevated BP levels, along with increased HR, suggest a disruption of sympathetic and parasympathetic input to the heart, which may be regarded as a cardiovascular risk factor. Previous studies have indicated the role of baroreflex disruption in the emergence of heart diseases (29). In the present study, treatment with E_2_ efficiently attenuated the altered baroreflex sensitivity in the OVX group. Additionally, HR, SBP, DBP, and MAP values also improved slightly in the OVX+E_2_ group compared with the OVX group (Figure 1). These observations are in line with another study (30), which indicated a potential for estrogen to mask the rise in BP and HR. Reflex bradycardia evoked by intravenous injection of phenylephrine has been well documented as a reliable measure of parasympathetic tone, while nitroprusside-induced reflex tachycardia is an indicator of sympathetic tone (31,32). In our study, the OVX group had a significant decrease in baroreflex sensitivity in response to phenylephrine and sodium nitroprusside when compared with the sham-operated group. In the OVX+E_2_ group, compared with the OVX group, treatment with E_2_ significantly improved baroreflex sensitivity. Based on these findings, we conclude that improved baroreflex sensitivity following estrogen administration is associated with a decrease in sympathetic tone, an attenuated pressure response to phenylephrine, and an enhanced pressor response, accompanied by an attenuated reflex tachycardia response to nitroprusside (33).

Oxidative stress and inflammation have been established as key drivers of CVDs (34). In the present study, MDA – a major lipid peroxidation end product and biomarker of oxidative stress – was found to be elevated in OVX rats (Figure 3). Similarly, SOD – a critical antioxidant enzyme and an indirect marker of oxidative stress – was also found to be reduced in OVX rats (Figure 3). These findings are aligned with those of previous studies and confirm the altered redox state in OVX rats (35). After treatment with E_2,_ MDA levels decreased slightly in OVX+E_2_ rats, while SOD levels increased significantly in OVX+E_2_ rats compared with OVX rats (Figures 3A and 3B), indicating that the antioxidative effects of estrogen may occur due to direct effects or indirectly by targeting cellular antioxidative defense mechanisms (36). In our study, all these oxidative stress parameters, including NO and SOD, were significantly reduced in the OVX group compared with the sham-operated group, indicating a state of oxidative stress in OVX rats. Treatment with E_2_ significantly improved plasma SOD and NO levels in the OVX-E_2_ group compared with the sham-operated group (Figure 3). Additionally, MDA levels increased significantly in the OVX group compared with the sham-operated group and decreased significantly following treatment with E_2_. We conclude from these findings that estrogen mediates antioxidative stress protection against CVDs, as previously reported in the literature (37).

As a critical molecule, NO plays a pleiotropic role in maintaining vascular homeostasis and cardiovascular health (38). We observed a marked reduction in plasma NO levels in OVX rats (Figure 3). A decrease in NO levels is often regarded as a cardiovascular risk factor, as it leads to impaired endothelial functions (39) and is associated with CVDs and risk factors such as hypertension, hyperlipidemia, type 2 diabetes, smoking, and atherosclerosis, along with being a strong predictive factor of atherosclerotic disease progression (40). Treatment with E_2_ effectively increased plasma NO levels in OVX+E_2_ rats, which may have occurred due to E_2_-mediated upregulation of the eNOS pathway (41,42). As the chief source of NO in the endothelium, eNOS controls BP levels by maintaining vascular tone (43,44). Also, NO has an anti-inflammatory role on the endothelium through its free radical scavenging capacity and its ability to upregulate the activity of antioxidant enzymes like MDA and SOD (45). Therefore, estrogen-mediated production of NO may have been responsible for the antioxidative and anti-inflammatory effects observed in our study. Previous studies have suggested a link between oxidative stress and autonomic regulation of the cardiovascular system (4). Hence, we suspect that estrogen-mediated NO production and the antioxidative NO effects attenuated the baroreflex sensitivity changes after ovariectomy in the animals in our study and improved the hemodynamic profile in OVX rats.

Previous evidence has shown NO to be a potent vasodilator and to play an important role in the protection against the onset and progression of CVD. Any change in the bioavailability of NO results in a loss of cardioprotective effects and, in some cases, may even lead to disease progression (46). We focused on NO oxidative stress markers in the present study because our research has revealed important effects of estrogen on CVDs. Previous studies have found that the decline in estrogen levels following menopause is associated with a rapid increase in CVDs due to heightened sympathetic tone and elevated oxidative stress. Our results in the OVX group are aligned with these observations. Conversely, in the OVX+E_2_ group, we observed a reduction in oxidative stress, which attenuated sympatho-excitation. This effect could be attributed to the downregulation of the NADPH oxidase (NOX) pathway and the upregulation of SOD protein expression (15). Moreover, studies have demonstrated that estrogen may regulate the transcript factor NF-κB, which is important in regulating NADHase expression (47). Therefore, it is possible that NO, SOD, and MDA are regulated by E_2_, which in turn is regulated by inflammatory markers.

Based on these findings, we conclude that estrogen (E_2_) modulates sterile inflammatory markers and baroreflex sensitivity in ovariectomized female Wistar rats via the NO pathway, which enhances cardioprotection in females.

We also observed increased levels of inflammatory cytokines (IL-6, TNF-α, and HMGB-1) in OVX rats (Figure 4). It has been reported that abnormally high serum levels of TNF-α – which is a major proinflammatory cytokine – are associated with an increased incidence of atherosclerosis and hypertension (48). Elevated concentrations of HMGB-1 and IL-6 have also been correlated with the occurrence of CVDs, including myocardial infarction and atherosclerosis (49-51). Levels of the inflammatory mediators TNF-α, IL-6,and HMGB-1 also improved significantly after E2 treatment in OVX+E2 animals (Figure 4). These results are consistent with those of previous studies (52,53) and are indicative of an anti-inflammatory effect of estrogen. As inflammation is deeply entangled with redox modulation (54,55), it is possible that the anti-inflammatory effects observed after E_2_ administration occurred due to the antioxidative potential of estrogen.

In conclusion, the model we used in the present study simulated a condition of ovarian hormone deficiency through bilateral ovariectomy in rats, which could lead to cardiovascular complications, along with changes in oxidative stress and sterile inflammatory biomarkers. These effects were mitigated by exogenous estrogen, highlighting the role of this hormone in maintaining cardiovascular health. Previous studies have demonstrated that estrogen can regulate the transcript factor NF-κB, which is important in regulating NADHase expression (56,57). Based on these studies and our findings, it is likely that estrogen regulates NO, SOD, and MDA levels.
